# Supplementary material for: The Smooth Transition From Many-Legged to Bipedal Locomotion—Gradual Leg Force Reduction and its Impact on Total Ground Reaction Forces, Body Dynamics and Gait Transitions
Source: Front Bioeng Biotechnol. 2022 Feb 4;9:769684. doi: 10.3389/fbioe.2021.769684 (PMC8855104; doi:10.3389/fbioe.2021.769684)
Supplement: Supplementary file 1 [file DataSheet2.pdf]

Work flow: leg interference model with defined ground force reduction

- setting parameters
  - duty factor (0.3; 0.5; 0.7)
  - step size of force decrease (0.25)
  - initial number of leg pairs (here 4 or 10)
  - alignment of the contact phases (TD, MS or TO)
  - contralateral phase of the pacemaker leg pair (here always 0.5)

- definition of the phase space (phase relation between adjacent ipsilateral legs θ: 0 to 1 in steps of 0.005)

- creation of a typical GRF of a single leg (cosine shape see methods)

- leg by leg stepwise application of reduction steps (by reducing the amplitude (paradigm i) or with shape maintenance (ii to iv). The last pair of legs was not affected.

- applying duty factors (the length of strides, i.e. stance + swing, was always 200 data points)

- adjusting alignment when contact durations in the force-reduced legs vary (paradigms ii - iv)

- concatenation of 20 strides for all legs

- application of ipsilateral phase shift (θ)

- application of contralateral phase shift for the legs of one side

- pruning the ends of the array were, owing to the phase shifts, not all legs have force data

- summing up all single leg forces

- dividing the sum by its mean, i.e. normalization onto body weight

- fft analysis of the resulting weight-normalised total vertical force

- detection of harmonics

- depiction of total force amplitudes over the phase space for the different duty factors and varying numbers of propulsive leg pairs (see Figs. 2-4 and Supplemental Fig. 1)
