# Supplementary material for: The Smooth Transition From Many-Legged to Bipedal Locomotion—Gradual Leg Force Reduction and its Impact on Total Ground Reaction Forces, Body Dynamics and Gait Transitions
Source: Front Bioeng Biotechnol. 2022 Feb 4;9:769684. doi: 10.3389/fbioe.2021.769684 (PMC8855104; doi:10.3389/fbioe.2021.769684)
Supplement: Supplementary file 2 [file DataSheet1.pdf]

Supplementary Material:

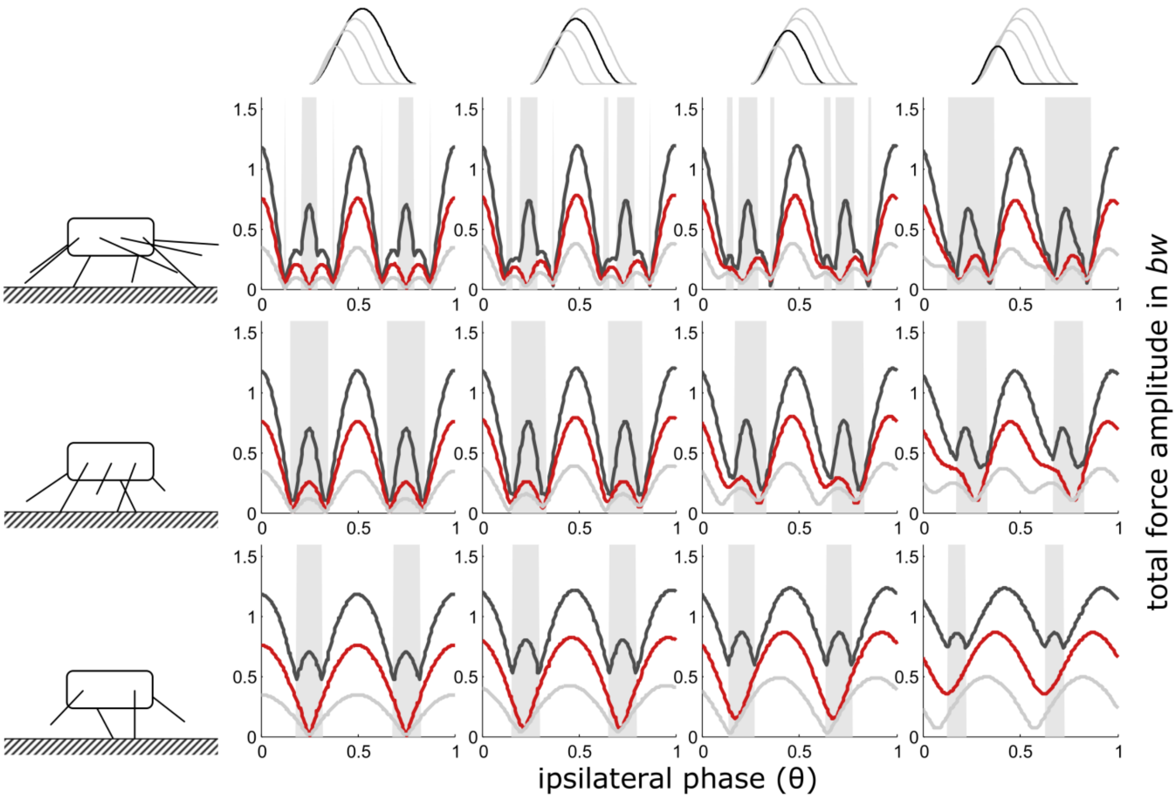

Fig. S1 Touch-down as reference point (ii): Dependency of total vertical leg force amplitudes on the phase shift of ipsilateral adjacent legs and duty factor with gradually decreasing contributions of increasing numbers of leg pairs. GRF decreased while their shape (ratio of width to height) remained constant i.e. also contact durations decreased with the reduction of the impulses beneath leg pairs. Duty factors shown: 0.3 (dark grey), 0.5 (red) and 0.8 (light grey). The top row indicates the degree of reduction in the pair of legs with reduced GRF and the type of force alignment (touch-down (ii)). Accordingly, in the third column from the right, the impulse beneath one leg pair is reduced by ¼, in the 2nd column from the right by ½ and in the rightmost column by ¾. The pictograms to the left indicate the number of propulsive pairs of legs valid for the respective row of subplots, i.e. four in the upper row, three in the middle row and two in the bottom row. With low duty factors and intermediate phase shifts, the peak frequency of the force oscillations assumed values higher than two times the stride frequency; these intervals are shaded in grey.

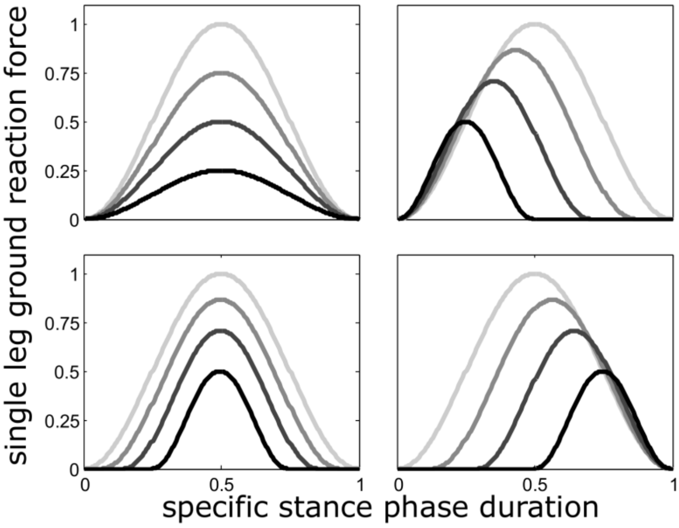

Fig. S2 Four different ways in which GRF reductions were implemented. Every force reduction step results in an impulse reduction of ¼. GRF reduction was implemented either by decreasing force amplitudes only (upper left; scheme i) or by a combined reduction of contact duration and force amplitude (schemes ii to iv; see methods). When GRF reduction followed the latter, reduced forces were aligned in three different ways to the GRF of the remaining legs. Reduced GRFs were aligned for θ = 0.5 either to touch-down (top right), mid-stance (bottom left) or take-off (bottom right) of the legs with normal GRF.

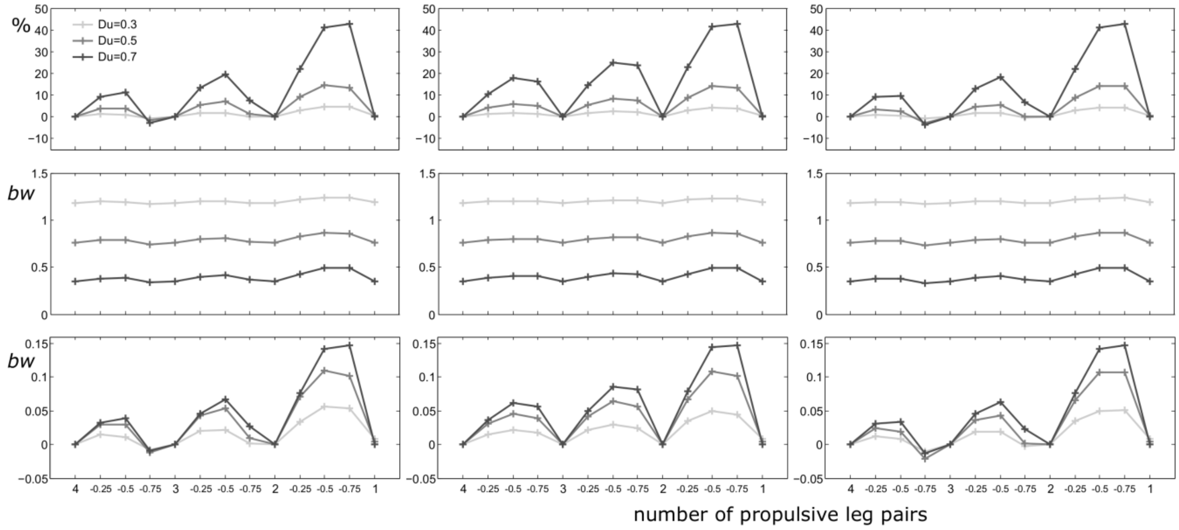

Fig. S3 Total force excesses for shape consistent force reduction. Left: take-off alignment (iv); Middle: mid-stance alignment (iii); Right: touch-down alignment (ii). The abscissa gives the number of propulsive pairs of legs (large numbers) and the degree of force reduction (small numbers). Results are shown for duty factors of 0.3, 0.5 and 0.7 (see legend). Upper row: relative force excess to integer numbers of leg pairs in %; second row: absolute maxima of the force amplitude values (at θ = 0, 0.5 or 1); bottom row: force difference in body weight.

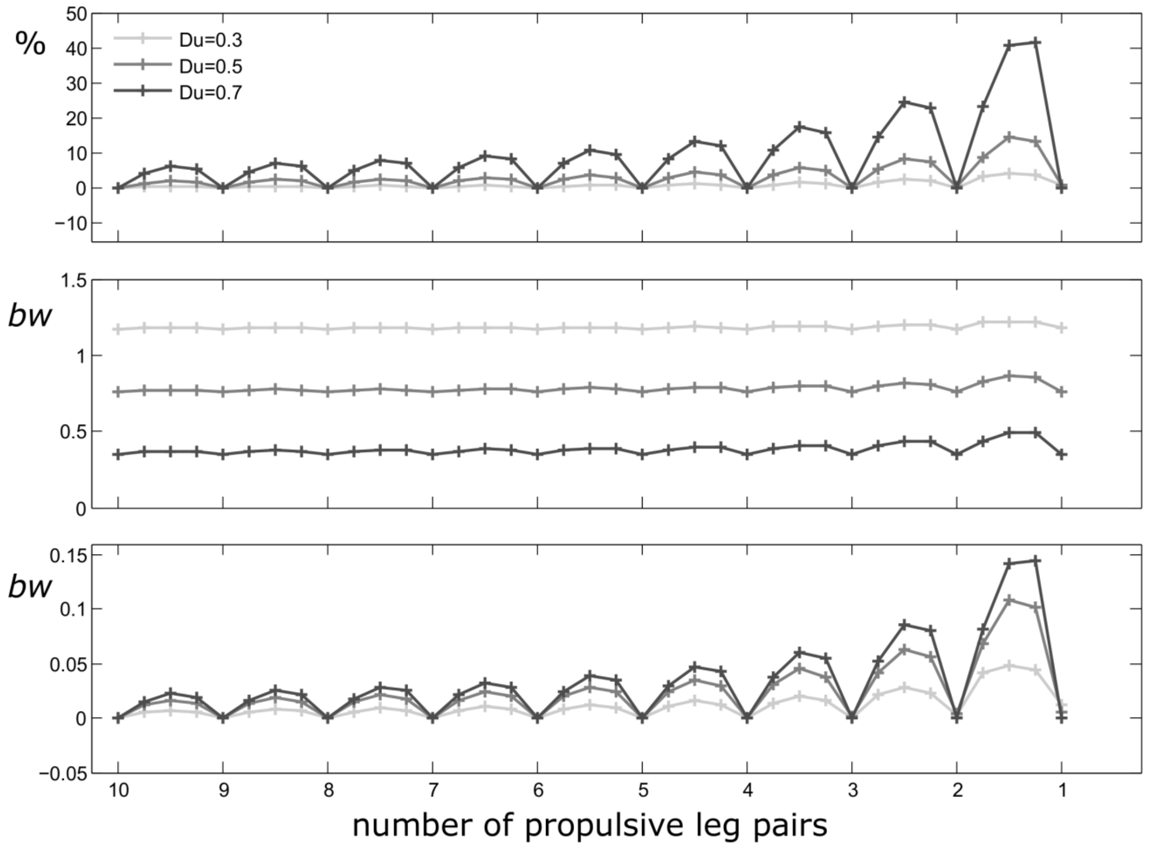

Fig. S4 Total force excesses for shape consistent force reduction and mid-stance alignment (scheme iii), for continuous GRF reductions from a locomotor apparatus with 10 pairs of legs down to only one pair of legs. The abscissa gives the number of propulsive pairs of legs. Results are shown for duty factors of 0.3, 0.5 and 0.7 (see legend). Upper row: relative force excess to integer numbers of leg pairs in %; second row: absolute maxima of the force amplitude values (at θ = 0, 0.5 or 1); bottom row: force difference in body weight.
